# Impact of estimated glucose disposal rate for identifying prevalent ischemic heart disease: findings from a cross-sectional study

**DOI:** 10.1186/s12872-022-02817-0

**Published:** 2022-08-20

**Authors:** Jin Xuan, Du Juan, Niu Yuyu, Ji Anjing

**Affiliations:** 1 Department of Cardiology, The Second Affiliated Hospital of Shandong, University of Traditional Chinese Medicine, Jinan, Shandong China; 2 Department of Cardiology, Rizhao Hospital of Traditional Chinese Medicine, Rizhao, Shandong China; 3 Department of Cardiology, Xinxiang First People’s Hospital, Xinxiang, Henan China; 4 Department of Cardiology, Yuhuan Second People’s Hospital, Huanbaozhong Road, Yuhuan, Zhejiang China

**Keywords:** Epidemiology, Estimated glucose disposal rate, Insulin resistance, Ischemic heart disease

## Abstract

**Background:**

Insulin resistance is one of the major mechanisms for cardiovascular events. Estimated glucose disposal rate(eGDR) has been demonstrated as a simple, accurate, and cost-effective estimator of insulin resistance. Our study aims to evaluate the correlation between eGDR and the prevalent IHD and assess the incremental value of eGDR for identifying prevalent IHD in the rural general population.

**Methods:**

Our study enrolled 10,895 participants from a cross-sectional survey of a metabolic management program. The survey was conducted in the rural areas of southeastern China between October 2019 and April 2020. eGDR = 21.158 − (0.09 * waist circumference) − (3.407 * hypertension) − (0.551 * HbA1c).

**Results:**

The prevalence of IHD was 4.20%. After adjusting for demographic, anthropometric, laboratory, and medical history covariates, each SD increase of eGDR brought a 25.9% risk reduction for prevalent IHD. After dividing eGDR into groups, the top group had a 58.9% risk reduction than the bottom group. Furthermore, smooth curve fitting demonstrated that the correlation between eGDR and prevalent IHD was linear in the whole range of eGDR. Additionally, AUC suggested that eGDR could significantly improve the identification of prevalent IHD by adding it to cardiovascular risk factors (0.703 vs. 0.711, *P* for comparison = 0.041). Finally, the category-free net reclassification index and integrated discrimination index also implicated the improvement from eGDR to identify prevalent IHD.

**Conclusion:**

Our data demonstrated a significant, negative, and linear correlation between eGDR and prevalent IHD. Our findings could suggest the potential usefulness of eGDR to improve the identification of prevalent IHD in the rural general population.

## Introduction

Even with the condition of intensive medical care, ischemic heart disease (IHD) is still one of the dominant causes of mortality worldwide. The attributed death caused by IHD was 116.9 per 100,000 worldwide in 2017 [1]. Similarly, mortality from IHD in China also reached 124 per 100,000 in 2017, and the data displayed a 20.6% increase compared with that in 1990 [2]. Although the electrocardiogram is a readily available, cheap, and accurate examination, the correct identification of IHD (especially the stable IHD) through electrocardiogram requires systemic learning and long-term experience accumulation, which are currently absent for the village doctor in the rural areas of China. Accordingly, an objective and quantified parameter or indicator could be more friendly for them to facilitate and simplify the identification of IHD in the rural general population.

Insulin resistance (IR) is one of the primary mechanisms for cardiovascular events [3]. As a central mechanism, IR links together all components of metabolic syndrome, including hypertension, dyslipidemia, hyperglycemia, and central obesity, which are major risk factors for cardiovascular events [4]. Moreover, prior studies have demonstrated that IR itself is also an independent risk factor for IHD [5–7]. On the contrary, a published article has revealed that the prevention of IR could diminish the probability of myocardial infarction by about 42% in a population of young adults via a mathematical analysis [8]. Data from the lab have also demonstrated that IR and the following hyperinsulinemia could accelerate the development of cardiovascular events via stimulating vascular stiffness, promoting atherosclerotic plaque formation, enhancing thrombosis, inhibiting fibrinolysis, and maintaining the persistence of low-grade inflammation [3, 9]. Therefore, estimating the degree of IR could benefit the early identification of IHD. However, the current gold standard for IR requires specialized equipment, which is rarely available in primary care conditions. Accordingly, a simple, economical, and non-invasive method to achieve routine monitoring of IR is needed to improve the early identification of IHD in the rural general population.

Estimated glucose disposal rate (eGDR) was proposed to estimate the severity of IR [10, 11]. eGDR has been demonstrated to have high precision in estimating IR compared to the euglycemic hyperinsulinemic clamp approach [12]. Additionally, published data have revealed the usefulness of eGDR in predicting cardiovascular events in diabetic patients [13–16]. However, evidence about the association between eGDR and the prevalent IHD in the general population is still limited. Hence, our current analysis aims to evaluate the association between eGDR and the prevalent IHD and assess the value of eGDR to improve the identification of prevalent IHD in a rural general population.

## Methods

### Study population

**Fig. 1:**
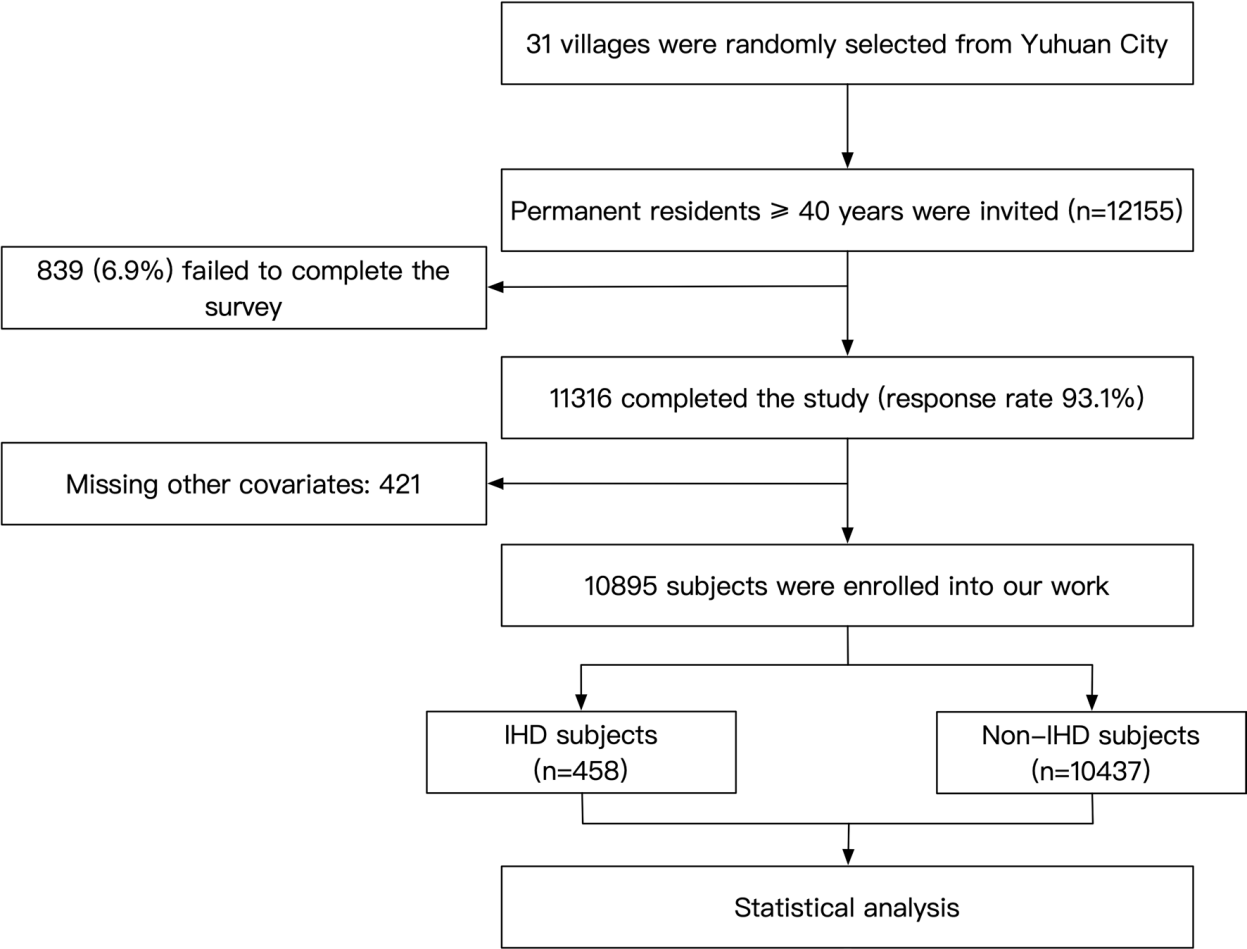
Flow chart of the enrolling process

The National Metabolic Management Center (MMC) is a metabolic care system conducted in Chine since 2016. Based on advanced medical equipment and telecommunication, it was funded to establish a standard and reproducible platform for diagnosing and managing metabolic disorders. Our current study derived from a branch cross-sectional survey of the MMC. The survey was conducted in the rural areas of eastern Zhejiang between October 2019 and April 2020. The survey adopted a clustered random sampling method. In general, 31 villages from Yuhuan city were randomly selected, and all permanent residents aged ≥ 40 years old without any exclusion criterion were enrolled into our survey. The exclusion criteria included cancer, mental disorder, and pregnancy. Eventually, 11,316 subjects completed the survey. An additional 421 subjects were excluded in the present work due to missed data of covariates, and 10,895 subjects were finally enrolled into our current work (Fig. 1). The central ethics committee of Yuhuan second People’s Hospital approved the study protocol of the survey, and the survey was conducted on the base of the Declaration of Helsinki.

### Data collection and measurement

A team of medical students, epidemiologists, general practitioners, cardiologists, and neurologists was employed to conduct the data collection. All the members underwent an epidemiological course, passed a final examination, and were authorized to collect data. The study was conducted in clinics with large rooms designed for primary medical care by the local health commission.

The medical staff filled out a standardized questionnaire for every subject during a clinical visit. For demographic data, education level was defined into three groups: primary school or below, middle school, and high school or above. Frequent exercise was defined as an average of more than three hours of mild sweating activity per day. Family annual income was summarized into three levels: less than 5000 Chinese Yuan (CNY), 5000–20,000 CNY, or more than 20,000 CNY. Current smoking was determined as smoking more than 100 cigarettes within the past one year. Current drinking was defined as drinking an alcoholic beverage equal to or more than five times in the past one year.

Subjects were asked to wear light clothes and take off their shoes when measuring their anthropometric parameters. Weight and height were quantified into the nearest 0.1 kg and 0.1 cm by calibrated electric scales and stadiometers when the subjects were standing. Meanwhile, waist circumference (WC) was recorded at 1 cm above the umbilicus.

Blood pressure measurement was performed via calibrated electronic sphygmomanometers (HEM-7136; Omron, Kyoto, Japan). Measurement was conducted in the large, quiet room of the clinics. Subjects were required to avoid exercise, smoking, and caffeine intake within 30 min before the measurement. Every two measures had a two-minute interval between them. Three effective and consecutive recordings were used for each subject. The mean value of the three records was taken into our statistical analysis.

After about eight hours of fasting, fasting blood samples were collected from every subject. Blood samples were collected from the cephalic vein by veno-puncture and then stored in EDTA vacutainer tubes (Becton, Dickinson and CO., Franklin Lakes, NJ, USA). The samples were centrifugated in site to isolate the serum from the whole blood. Then the samples were stored at − 20 ℃. Subsequently, they were delivered to Yuhuan second people’s hospital for laboratory analysis. Blood biochemical indices were quantified enzymatically by an auto-analyzer (Abbott Laboratories, Abbott Park, Illinois, USA). In addition, about 5% of the blood samples were randomly selected and re-tested at a certified 3rd party institute to enhance the accuracy of the laboratory test.

Twelve-leads ECG (Jinjiang, LEAD-7000c) was conducted for every subject in resting position for at least 10 s. Two cardiologists were employed to conduct and analyze the ECGs using callipers and magnifying glasses.

### Definition

Anti-hypertensive therapy was determined as the usage of any anti-hypertensive drug in the past two weeks. Hypertension was defined as mean systolic blood pressure (mSBP) equal to or more than 140 mmHg and/or mean diastolic blood pressure (mDBP) equal to or more than 90 mmHg; subjects with self-reported anti-hypertensive therapy were also regarded as hypertensive patients [17]. Anti-diabetic therapy was referred to the intake of any anti-diabetic drug in the past two weeks. And diabetes was defined as fasting plasma glucose (FPG) equal to or more than 7 mmol/L and/or glycated hemoglobin (HbA1c) ≥ 6.5% and/or self-reported anti-diabetic therapy [18]. Obesity was defined as BMI ≥ 28 kg/m^2^ according to the Chinese guideline [19]. eGDR was calculated according to the following formula: eGDR = 21.158 − (0.09 * WC) − (3.407 * hypertension) − (0.551 * HbA1c). [hypertension (yes = 1/no = 0), HbA1c = HbA1c (%)] [11, 12]. Diagnosis of IHD relied on ECG results, medical records, and concrete medical examinations. Similar to the standard used in the Framingham study [20], copies of medical records for hospitalizations and outpatient cardiovascular diagnoses were obtained from subjects; two cardiologists reviewed the electrocardiogram and medical history independently to screen out the subjects suspect of IHD (ICD-10 code I 20 for angina pectoris, I 21 for acute myocardial infarction). For any subject suspected of having IHD, the cardiologists would further review the laboratory, imaging, and procedural data (including coronary computational tomography angiography and coronary angiography) from the corresponding hospitals. Only subjects with concrete evidence were diagnosed with IHD. To ensure the accuracy of the diagnosis, two additional cardiologists would review the controversial cases to discuss for the final diagnosis.

### Statistical analysis

Based on their distributions, continuous variables were shown as mean values (standard deviation, SD) or median (quartile 1–quartile 3). Categorical variates were summarized as frequency (percentage). The student’s *t* test tested the difference between continuous variates for normal distribution or the Mann–Whitney test for skewed distribution. The Chi-square test and the Rank-sum test were used to assess the difference of categorical variates and ordinal categorical variates between groups, respectively. When analyzed as a continuous variable, eGDR was natural log-transformed before being included into statistical analysis due to its skewed distribution. Normalization of eGDR was conducted by z-score [(eGDR-mean value of eGDR)/ standard deviation (SD) of eGDR]. Furthermore, eGDR was categorized into four groups according to previously used cut-off levels [12, 21]. Multivariate logistic regression analysis investigated the independent association between eGDR and prevalent IHD. The results were shown as odds ratios (ORs) and 95% confidence intervals (95% CI). To confirm whether the correlation between eGDR and the prevalent IHD was linear in the full range of eGDR, we employed a generalized additive model with a spline smoothing function. We also conducted the subgroup analysis to assess whether some common cardiovascular risk factors could influence the association between eGDR and the prevalent IHD. The grouping factors included sex, age (grouped into < 60 and ≥ 60 years), obesity, hypertension, and diabetes. Additionally, the present work conducted receiver operating characteristic (ROC) curve and reclassification analysis (includes category-free net reclassification index, NRI, and integrated discrimination index, IDI) to evaluate the value of eGDR for improving the identification of prevalent IHD. All the statistical analysis was conducted through statistical software packages R (http://www.R-project.org, The R Foundation), EmpowerStats (http://www.empowerstats.com, X&Y Solutions, Inc., Boston, MA), and SPSS 26.0 software (IBM Corp). Statistical significance was set as a 2-tailed *P* value less than 0.05.

## Results

The characteristics of the 10,895 enrolled participants were summarized in Table 1. The prevalence of IHD was 4.20%. Regarding the demographic data, IHD patients had a higher age level, lower education and income levels, a higher percentage of frequent exercise, and a lower percentage of current drinking. As for the anthropometric data, IHD patients had higher weight, BMI, WC, and blood pressure levels than non-IHD subjects. Similarly, laboratory data displayed that IHD patients had worse glucose and lipids conditions than their healthy counterparts. Moreover, IHD patients had higher rates of anti-hypertensive, anti-diabetic, and lipid-lowering therapy, and the prevalence of hypertension and diabetes were higher in the IHD group than in the non-IHD group. Finally, IHD patients also had a lower level of eGDR than non-IHD subjects.

**Table 1:** Characteristics of included participants

Variables	Total (n = 10,895)	IHD (458)	Non-IHD (n = 10,437)	*P* value
Age (years)	59.95 (10.07)	64.96 ± 8.17	59.73 ± 10.08	< 0.001
Male (%)	4379 (40.19)	175 (38.21)	4204 (40.28)	0.376
Education level (%)				0.005
Primary school or below	6445 (59.16)	302 (65.94)	6143 (58.86)	
Middle school	3483 (31.97)	115 (25.11)	3368 (32.27)	
High school or above	967 (8.88)	41 (8.95)	926 (8.87)	
Income (CNY) (%)				< 0.001
≤ 5000	4716 (43.29)	285 (62.23)	4431 (42.45)	
5000–20,000	4331 (39.75)	124 (27.07)	4207 (40.31)	
> 20,000	1848 (16.96)	49 (10.70)	1799 (17.24)	
Frequent exercise (%)	1806 (16.58)	166 (36.24)	1640 (15.71)	< 0.001
Current smoking (%)	2881 (26.44)	108 (23.58)	2773 (26.57)	0.156
Current drinking (%)	3065 (28.13)	90 (19.65)	2975 (28.50)	< 0.001
Height (cm)	159.38 (8.04)	158.81 ± 7.73	159.41 ± 8.05	0.117
Weight (kg)	63.17 (11.34)	64.19 ± 12.40	63.12 ± 11.29	0.048
BMI (kg/m²)	24.81 (3.77)	25.38 ± 4.17	24.79 ± 3.75	< 0.001
WC (cm)	83.39 (10.22)	85.49 ± 11.87	83.29 ± 10.13	< 0.001
SBP (mmHg)	145.71 (23.62)	153.68 ± 23.60	145.36 ± 23.56	< 0.001
DBP (mmHg)	86.69 (11.93)	87.90 ± 12.25	86.64 ± 11.91	0.027
FPG (mmol/L)	5.74 (5.25–6.37)	6.05 (5.49–6.88)	5.73 (5.24–6.35)	< 0.001
HbA1c (%)	5.40 (5.10–5.80)	5.50 (5.10-6.00)	5.40 (5.10–5.80)	0.001
TC (mmol/L)	5.02 (4.38–5.74)	5.12 (4.35–5.98)	5.01 (4.38–5.73)	0.151
TG (mmol/L)	1.29 (0.91–1.89)	1.41 (1.00-1.99)	1.29 (0.90–1.89)	< 0.001
HDL-C (mmol/L)	1.79 (1.38–2.43)	1.50 (1.23–1.98)	1.80 (1.39–2.45)	< 0.001
LDL-C (mmol/L)	2.18 (1.51-3.00)	2.50 (1.82–3.10)	2.16 (1.50-3.00)	< 0.001
Anti-hypertensive therapy (%)	2337 (21.45)	230 (50.22)	2107 (20.19)	< 0.001
Anti-diabetic therapy (%)	579 (5.31)	55 (12.01)	524 (5.02)	< 0.001
Lipid-lowering therapy (%)	215 (1.97)	43 (9.39)	172 (1.65)	< 0.001
Hypertension (%)	6602 (60.60)	363 (79.26)	6239 (59.78)	< 0.001
Diabetes (%)	1767 (16.22)	126 (27.51)	1641 (15.72)	< 0.001
eGDR	8.04 (6.78–10.62)	7.21 (6.03–8.74)	8.08 (6.81–10.66)	< 0.001

Data were displayed as mean (SD), median (quartile 1–quartile 3), and numbers (percentage) according to their data type and distribution

*IHD* ischemic heart disease, *CNY* Chinese currency, *BMI* body mass index, *WC* waist circumstance, *SBP* systolic blood pressure, *DBP* diastolic blood pressure, *FPG* fasting plasma glucose, *HbA1c* glycated hemoglobin, *TC* total cholesterol, *TG* triglyceride, *HDL-C* high-density lipoprotein cholesterol, *LDL-C* low-density lipoprotein cholesterol, *eGDR* estimated glucose disposal rate

The logistic regression revealed a significant and negative association between eGDR and the prevalent IHD; the results were displayed in Table 2. Without any adjustment, the risk of prevalent IHD decreased by 32.6% for every SD increment of eGDR. After adjusting for age, gender, education, income, and physical activity levels, current smoking and drinking conditions, each SD increase of eGDR only resulted in a 27.4% decrease of the risk of prevalent IHD (Model 1). After further adjustment of BMI, WC, TC, HDL-c, hypertension, diabetes, and lipid-lowering therapy, each SD increase of eGDR could only decrease the risk of prevalent IHD by 25.9%. We further divided eGDR into groups according to the standard in published articles. The prevalence of IHD was 8.5% in the lowest group, 9.4% in the second group, 4.5% in the third group, and 2.8% in the highest group. The 4th group had a 32.2% risk of prevalent IHD compared to the 1st group in the crude model. In model 1, the risk of prevalent IHD in the top group increased to 40.0% compared with the bottom group. In the fully adjusted model, the risk of prevalent IHD in the top group further inflated to 41.1% compared with the bottom group. Furthermore, we observed a significant trend towards a lower risk of prevalent IHD across the groups (all *P* for trend < 0.001).

**Table 2:** Multivariate logistic regression evaluating the correlation between eGDR and prevalent IHD

Variables	Prevalence of IHD (%)	Odds ratio (95% CI)					
		Crude	*P* value	Model 1	*P* value	Model 2	*P* value
eGDR (per 1 SD increase)	–	0.674 (0.619, 0.733)	< 0.001	0.726 (0.664, 0.794)	< 0.001	0.741 (0.636, 0.864)	< 0.001
Groups of eGDR							
eGDR < 4	11 (8.5)	Reference		Reference		Reference	
4 ≤ eGDR < 6	103 (9.4)	1.135 (0.593,2.174)	0.702	1.140 (0.589, 2.203)	0.698	1.155 (0.586, 2.276)	0.677
6 ≤ eGDR < 8	187 (4.5)	0.520 (0.276, 0.980)	0.043	0.557 (0.292, 1.060)	0.075	0.582 (0.285, 1.189)	0.138
8 ≤ eGDR	157 (2.8)	0.322 (0.170, 0.609)	< 0.001	0.400 (0.209, 0.766)	0.006	0.411 (0.189, 0.896)	0.025
*P* for trend	–		< 0.001		< 0.001		< 0.001

Crude model: no adjustment; Model 1: adjusted for age, sex, education level, income level, physical activity, current smoking, current drinking; Model 2: Model 1 + BMI, WC, TC, HDL, hypertension, diabetes, and lipid-lowering therapy

*eGDR* estimated glucose disposal rate, *IHD* ischemic heart disease, *CI* confidence interval, *SD* standard deviation, *BMI* body mass index, *WC* waist circumference, *TC* total cholesterol, *HDL-C* high-density lipoprotein cholesterol

**Fig. 2:**
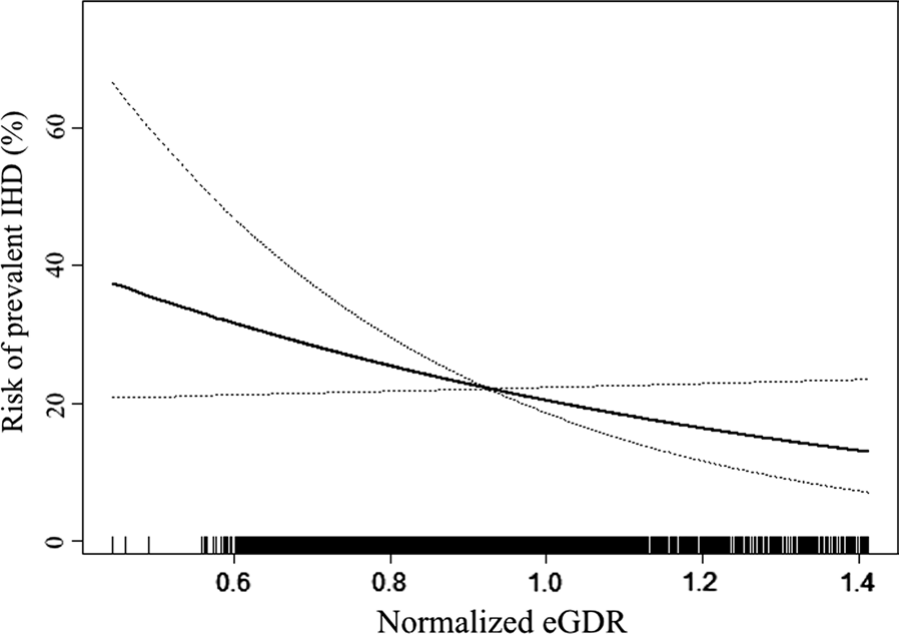
Smooth curve fitting assessing the correlation between eGDR and the risk of the prevent IHD. Smooth curve fitting was conducted through generalized addictive model and adjusted for all covariates used in Model 2 of Table 2. In the figure, the risk of prevalent IHD declined proportionally with the increment of eGDR, implicating the correlation between eGDR and prevalent LVH was linear in the whole range of eGDR.

Our work further employed smooth curve fitting to verify the linear trend observed in Table 2; the result was displayed in Fig. 2. The plot demonstrated that the risk of prevalent IHD decreased proportionally with the increase of eGDR. Therefore, the correlation between eGDR and the prevalent IHD was linear in the whole range of eGDR.

**Fig. 3:**
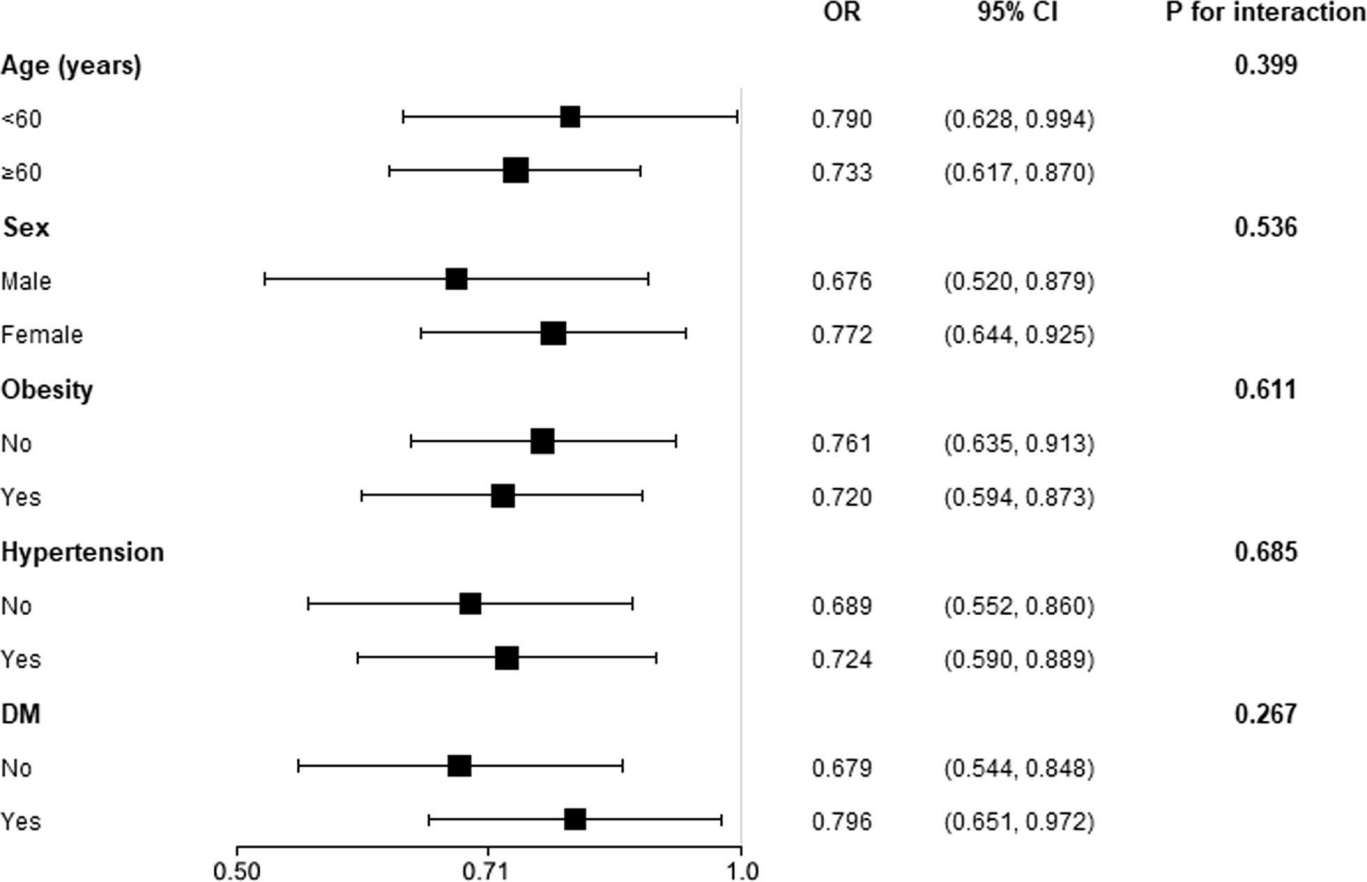
Subgroup analysis of the association between eGDR and prevalent IHD The model in each stratum was adjusted of all covariates used in Model 2 of Table 2, except for the variate that was used to define subgroups. P for interaction in all subgroups was insignificant, suggesting the association between eGDR and prevalent IHD was robust in these sub-populations

Our current work also performed the subgroup analysis to evaluate whether the main finding from the logistic regression was robust in some common sub-populations (Fig. 3). The subgroups in our study included sex, age, obesity, diabetes, and hypertension. The logistic regression models in the subgroup analysis were adjusted for all covariates used in our main results (Model 2 of Table 2), except for the variate that was used to define subgroup. The results demonstrated that our major finding from the entire population was robust in these subgroups (all *P* for interaction > 0.05).

To assess the potential usefulness of eGDR to identify the prevalent IHD in the general population, we further conducted ROC and reclassification analysis; the results were shown in Table 3. The AUC of eGDR alone for identifying the prevalent IHD was 0.620 (95% CI 0.611–0.629, *P* < 0.001). After introducing eGDR into established IHD risk factors (age, gender, current smoking and drinking conditions, BMI, WC, TC, HDL-c, hypertension, diabetes, and lipid-lowering therapy), the results displayed a significant improvement in the identification of prevalent IHD (AUC: 0.703 vs. 0.711, *P* for comparison = 0.041). Additionally, category-free NRI (0.209, 95% CI 0.121–0.297, *P* < 0.001) and IDI (0.004, 95% CI 0.002–0.007, *P* < 0.001) also displayed significant values for eGDR to improve the identification of the prevalent IHD.

**Table 3:** ROC and reclassification analysis investigating the usefulness of eGDR to optimize the identification of prevalent IHD

Model	AUC (95% CI)	*P* value	*P* for comparison	NRI (category free)	*P* value	IDI	*P* value
eGDR	0.620 (0.611, 0.629)	< 0.001	–	–	–	–	–
Clinical risk factors*	0.703 (0.695, 0.712)	< 0.001	–	–	–	–	–
Clinical risk factors + eGDR	0.711 (0.703, 0.720)	< 0.001	0.041	0.209 (0.121, 0.297)	< 0.001	0.004 (0.002, 0.007)	< 0.001

*Clinical risk factors: age, sex, current smoking, current drinking, BMI, WC, TC, HDL, hypertension, diabetes, and lipid-lowering therapy

*ROC* receiver operating characteristic curve; *eGDR* estimated glocuse disposal rate; *IHD* ischemic heart disease; *AUC* area under the curve, *CI* confidence interval; *NRI* net reclassification improvement, *IDI* integrated discrimination index, *BMI*: body mass index, *WC* waist circumference, *TC*: total cholesterol, *HDL-C*: high-density lipoprotein cholestero

## Discussion

Our current work demonstrated a significant, negative, and linear correlation between eGDR and prevalent IHD, implicating a positive and significant correlation between the degree of insulin resistance and prevalent IHD. Furthermore, our study demonstrated the correlation between eGDR and prevalent IHD was robust in several common subpopulations. Additionally, our analysis revealed a significant improvement in identifying prevalent IHD when adding eGDR into several cardiovascular risk factors. The present analysis could implicate a potential correlation between eGDR, and the underlying insulin resistance, and the risk of the prevalent IHD. Furthermore, our study could also suggest the potential value of eGDR in improving the identification of prevalent IHD.

Our findings confirmed our hypothesis about the association between eGDR and prevalent IHD. After adjusting for demographic, anthropometric, laboratory, and medical history data, the logistic regression revealed a negative and significant correlation between eGDR and the prevalent IHD. Every SD increase of eGDR could diminish the risk of prevalent IHD by 25.9%. Because of the negative relationship between eGDR and the severity of IR, our data implicate the positive correlation between IR and the risk of the prevalent IHD. Moreover, by dividing eGDR into groups according to published criteria, we observed a linear trend towards a higher risk of the prevalent IHD across groups. Additionally, the smooth curve fitting demonstrated that the correlation between eGDR and the prevalent IHD was linear in the whole range of eGDR, implicating the risk decreases proportionally with the increment of eGDR. Therefore, eGDR may act as a linear indicator for the risk of the prevalent IHD. Finally, the results from the subgroup analysis implicate that the negative association between eGDR and the prevalent IHD is not influenced by sex, age, obesity, hypertension, and diabetes. Therefore, applying our primary result to these sub-populations could be reasonable and feasible.

To assess the value of eGDR to improve the identification of prevalent IHD in the general population, our study employed ROC and reclassification analysis. As for ROC analysis, eGDR alone had a moderate AUC, suggesting the limited value of eGDR alone to identify the prevalent IHD. However, by adding eGDR to several cardiovascular risk factors, we observed a significant improvement in identifying the prevalent IHD (AUC: 0.703 vs. 0.711, P for comparison = 0.041). Nevertheless, although ROC analysis is the most popular approach to assessing the value of a novel marker, ROC analysis still has its disadvantage. ROC analysis has a low sensitivity to recognize the usefulness of a novel marker to improve the identification of prevalent diseases [22]. ROC analysis focuses on the comparison of the ability of models to identify the risk of prevalent diseases, but it pays little attention to whether adding a new marker into existed risk factors could improve the identification of prevalent diseases [23]. Therefore, ROC analysis alone may not be able to evaluate the usefulness of a new marker to identify prevalent IHD comprehensively. Accordingly, scientists have begun to use reclassification analysis to investigate the incremental value of new markers to identify prevalent diseases [24–26]. In the current analysis, we also employed reclassification analysis to assess the value of eGDR. Both category-free NRI and IDI were significant, suggesting the potential incremental value of eGDR to improve the identification of the prevalent IHD. Therefore, both ROC and reclassification analysis bolstered the potential incremental value of eGDR. Hence, general practitioners in rural primary care conditions could achieve more precise identification of prevalent IHD than ever before by applying eGDR to daily clinical practice.

It is our obligation to elucidate the significance of applying eGDR to facilitate the identification of the prevalent IHD. In China, although the doctors from hospitals in large cities are well-educated and specialized, the primary practitioners, especially the village doctors in rural areas, still lack systemic medical education [27]. Due to the low income, village doctor is usually occupied by middle-aged people with the lowest medical education. Therefore, most of them did not receive systematic electrocardiogram training; thereby, they are unable to identify IHD, especially the stable IHD, independently. Due to this condition, an objective and quantified parameter or indicator could be more friendly for them to facilitate the identification of prevalent IHD in the rural population. From this view and combining our findings in the current study, we believe the village doctor in the rural areas of China could calculate eGDR to facilitate the identification of prevalent IHD in the rural general population.

When interpreting our findings, it is necessary to mention that the prevalence of IHD was relatively lower than those in similar surveys. Nevertheless, according to a published Chinese national survey of cardiovascular diseases, the prevalence of IHD in Chinese females and males was 0.51% and 0.74%, respectively [28]. The survey revealed that the prevalence of IHD in subjects aged ≥ 40 years old ranged from 0.28 to 2.41%. Moreover, the 2018 China cardiovascular diseases report demonstrated that the prevalence of IHD maintained at this level in the past ten years [29]. Additionally, the prevalence of IHD in our survey was similar to the prevalence in several Chinese cohorts [30–32]. Therefore, we believe the results from our study were still reliable but needed to be confirmed in other populations.

From the description of the characteristic data in Table 1, we observed that the IHD group had an approximately 80% prevalence of hypertension, and the median glycated hemoglobin level was 5.50%. This phenomenon may indicate that eGDR may predominantly depend on the presence of hypertension rather than diabetes. However, when comparing our results with the results from existing literatures, we found our observation is consistent with previous findings. In Penno et al.’s study, subjects with eGDR < 4.14 had a hypertension prevalence of 99.5%, subjects with eGDR value between 4.15 and 5.34 also had a hypertension prevalence of 98.7%, and subjects with eGDR ≥ 5.34 had a hypertension prevalence of 52.8% [11]; In their work, they also used euglycemic hyperinsulinemic clamp test to exam the correlation between eGDR and the level of measured glucose disposal rate, the results demonstrated that the correlation of eGDR with measured glucose disposal rate was highly significant (r = 0.624, *P* < 0.001). In another study assessing the value of eGDR, the prevalence of anti-hypertensive therapy reached 97.9% in subjects with eGDR < 4; On the contrary, the prevalence of anti-hypertensive therapy was only 41.0% and 1.1% in subjects with eGDR 6–8 and > 8, respectively [13]. In this research, the researcher also observed a close correlation between eGDR and the results of euglycemic hyperinsulinaemic clamp (r = 0.73). From the view of mechanisms, insulin resistance could lead to hypertension through multiple mechanisms; and multiple studies have identified the association between insulin resistance and the exacerbated risk of hypertension in the general population [33]. Based on the above information, we believe the high prevalence of hypertension in the IHD group, which had a low eGDR level, could still be reasonable, but further studies are still needed to assess whether the high prevalence of hypertension will influence the accuracy of eGDR to estimate the actual level of insulin resistance.

It is essential to notice the limitations in our current work when interpreting our findings. Firstly, our study originated from cross-sectional data of an epidemiological survey. Due to the pandemic of COVID-19, the follow-up data is unavailable until now. Therefore, our results only suggest the association between eGDR, and the underlying IR, and the prevalent IHD. The longitudinal relationship still needs prospective cohorts to confirm. Secondly, our subjects were sampled from a natural population in southeastern China. Hence, the findings from our work may not be applicable to people of different races and socioeconomic conditions and live in different geographic conditions; In other words, the external validity of the current findings still needs further studies to verify. Thirdly, the definition of IHD used in our work could overlook the asymptomatic obstructive IHD in the population. This is a major limitation of our manuscript. However, as an observational epidemiologic study with a relatively large sample size, it is not feasible to perform coronary computational tomograph or coronary angiography for all subjects. Studies with optimized definitions of IHD which can identify asymptomatic obstructive IHD are warranted to verify our conclusions. Lastly, because of the observational design of our survey, some unrecorded covariates may cast residual confounding and introduce bias into our results. For example, we measured the renal function only in a part of the participants. In the current analysis, most subjects were lack of renal function data. If we adjusted renal function in the current analysis, the sample size would shrink significantly, leading to a lack of statistical power. Accordingly, more longitudinal studies with more comprehensive and complete information collection are needed to verify the association between eGDR and the risk of prevalent IHD.

## Conclusion

In general, our work demonstrated a negative, linear, and robust association between eGDR, a simple surrogate of IR, and the risk of the prevalent IHD in a rural general population. Moreover, our data could suggest the potential value of eGDR to improve the identification of the prevalent IHD in the general population, implicating the potential usefulness of eGDR to serve as a rapid, simple, and cost-effective marker to improve the identification of prevalent IHD in rural areas.

## Data Availability

The datasets generated and/or analyzed during the current study are not publicly available currently because the survey is part of an ongoing cohort, which has a limited access period due to the regulation of local technology bureau. However, the datasets will be available from the corresponding author on reasonable request.
